# Thermo-/pH-Dual-Sensitive PEG/PAMAM Nanogel: Reaction Dynamics and Plugging Application of CO_2_ Channeling

**DOI:** 10.3390/gels8100683

**Published:** 2022-10-21

**Authors:** Xiangbin Liu, Suling Wang, Weiguang Shi, He Liu

**Affiliations:** 1 School of Mechanical Science and Engineering, Northeast Petroleum University, Daqing 163453, China; 2 Oil Production Engineering Research Institute of Daqing Oilfield Ltd., Daqing 163453, China; 3 Key Laboratory of Continental Shale Hydrocarbon Accumulation and Efficient Development, Ministry of Education, Northeast Petroleum University, Daqing 163453, China; 4 Provincial Key Laboratory of Oil and Gas Chemical Technology, College of Chemistry and Chemical Engineering, Northeast Petroleum University, Daqing 163318, China; 5 PetroChina Research Institute of Petroleum Exploration & Development, Beijing 100083, China

**Keywords:** smart nanogel, PEG, PAMAM, reaction dynamics, CO_2_ channeling plugging

## Abstract

Smart hydrogels, owing to their exceptional viscoelastic and deformable capacity in response to environmental stimulation involving temperature and pH, have been successfully applied in oilfields for purposes such as water and/or gas shutoff treatments. However, the CO_2_ breakthrough problem in low permeability reservoirs has not been well solved. In this work, a rheological method-based Avrami dynamics model and Dickinson dynamics model were employed to investigate the dynamic gelation process of thermo-/pH-dual-sensitive PEG/PAMAM nanogels to further our understanding of the microstructure of their gelation and pertinence plugging application. Plugging experiments were performed by alternating injections of CO_2_ and hydrogel solution in a slug type on three fractured low permeability cores with a backpressure of 13 MPa. The nanogels presented a secondary growth pattern from three to one dimension from micrometer to nanometer size with a morphological transformation from a sphere to an irregular ellipsoid or disk shape. The phase transition temperature was 50 °C, and the phase transition pH was 10. If both or either were below these values, the hydrogel swelled; otherwise, it shrank. Plugging results show that the plugging efficiency was higher than 99%. The maximum breakthrough pressure was 19.93 MPa, and the corresponding residual pressure remained 17.64 MPa for a 10 mD core, exhibiting great plugging performance and high residual resistance after being broken through by CO_2_.

## 1. Introduction

Environmental stimulus-responsive hydrogels exhibit magnificent features, including reversible swell and shrink behavior, novel mechanical properties, and special sensitivity in response to light, electric or magnetic fields, temperature, pressure, pH, ionic strength, and specific molecules [1,2,3]. These smart hydrogels are increasingly attracting more and more interest for marvelous applications, such as bioengineering, sensors, molecular separation, drug delivery, and reservoir plugging in oilfields [4,5,6,7]. In many practical cases, temperature and pH, two important environmental factors, may occur at the same time in biological, chemical, and/or geoscience systems [8,9,10]. Therefore, thermo-/pH-dual-responsive hydrogels have received increasing attention.

Generally, molecular structures with temperature- and pH-responsive groups in the smart hydrogels are the key issues that must be addressed to perform the swelling and deswelling behaviors. The physical and/or chemical crosslinking of hydrogels in the swollen state can be tuned by hydrophobic groups (–nCH_2_–, –nCH_2_-CH_2_-O–, and –nSi-O–) and hydrophilic groups (–NH_2_, –COOH, –OH, –CONH–, and –SO_3_H), which can control the volume changes and the sol–gel transition [11]. Recently, novel strategies have been developed to improve the response rate of thermo-/pH-dual-responsive hydrogels via crosslinking reactions between poly(amido amine) (PAMAM) dendrimers and poly(ethylene glycol) (PEG) [12,13]. In most PAMAM-PEG hydrogels, PAMAM with amino-terminated structures exhibits pH sensitivities owing to the primary amines and tertiary amines, and PEGs with linear chains achieve temperature controllability via strong hydrophilic performance and hydrophobic effects [14,15]. Thus, PAMAM-PEG hydrogels have attracted widespread attention due to their non-toxic, non-polluting, biocompatible, and environmentally friendly properties.

Hydrogels are 3D cross-linked network polymers, and physical and structural changes affect their microstructures and functional properties. Therefore, kinetic research can provide insights into the formation process of gels. Previous studies have reported that two growth modes of gel networks (bulb growth mode and fiber growth mode) can control the gel properties [16]. The Avrami model has been widely used to analyze the dynamics of small and macromolecular polymers, in which the obtained kinetic parameters are closely related to the growth dimension and the nucleation mechanism, establishing a relationship between dynamics and gel structures [17,18]. According to the difference in the Avrami factor, the process of fiber network structure is studied from one-dimensional growth to the whole fiber network. However, a comprehensive understanding of the reaction kinetics of spherical hydrogels, especially with temperature- and pH-responsivity, remains a great challenge.

In the meantime, Hydrogels exhibit complex rheological behaviors, such as elasticity and viscosity. The elastic modulus, energy storage modulus, and loss modulus are closely related to the network structure of the gel. Recently, a variety of experimental methods have been employed to prove that there is an inevitable relationship between the structure and dynamics of polymer networks and their rheological behavior [19,20]. The rheological study results of static and dynamic polymer networks demonstrate that 3D cross-linked structures and micelle structures can strongly influence the rheological properties [19]. Nevertheless, a clear understanding of the relationship between the nano spherical structure and reaction dynamics of temperature- and pH-responsive hydrogels is still challenging.

In the petroleum industry, functional and smart hydrogels have been developed to increase conformance, reduce the channeling from fractures and voids in water and gas flooding, increase oil recovery, and reduce water production [21,22]. Nowadays, it has been proven that wormlike micelles with amidino groups, polyethyleneimine, and polyacrylamide-based polysaccharide sealants have a CO_2_-responsive capacity to seal a gas leakage pathway [23,24,25]. However, due to the large size of hydrogel particles, the transport distance in the formation is limited, and so they cannot meet the requirements of in-depth profile control or oil displacement in oil–gas reservoirs. In the late stage of CO_2_ flooding in particular, although CO_2_ flooding is an effective technology for low permeability reservoir development, the gas channeling problem is serious in low permeability reservoirs [26,27]. Thus, there is an urgent need for novel thermo-/pH-dual-responsive nanogel designs that can overcome the size-matching difficulty, maintain the environmental thixotropic stability, and improve the plugging efficiency in low-permeability CO_2_ flooding reservoirs.

In this work, the thermo-/pH-dual-sensitive nanostructured PEG/PAMAM hydrogels synthesized in our previous report were employed to research the reaction dynamics and application of plugging CO_2_ channeling [28]. Firstly, the rheological method was used to study the fluidity and elasticity information of the intelligent gel, obtaining the quantitative relationship between the dynamic gelation process and the composite modulus. Secondly, the Avrami kinetic model was employed to calculate the correlation between the composite viscoelastic modulus and the Avrami factor in order to obtain the nucleation and growth modes. Thirdly, the Dickinson model was used to study the relationship between gel aggregation state (D-density dimension parameter) and microstructure [29]. Finally, the reaction dynamics were established for the thermo-/pH-dual-sensitive nanostructured PAMAM-PEG hydrogel. Moreover, the physical simulation experiment was carried out to test the plugging performance of environmentally responsive hydrogel. This work will provide theoretical and technical support and explicit guidance to solve the practical problem of gas channeling and plugging in low-permeability CO_2_ reservoir flooding.

## 2. Results and Discussion

The thermo-/pH-dual-sensitive nanostructured PEG/PAMAM hydrogels were synthesized by the same method reported in our previous work [28]. The environmental response properties were studied, the gel formation kinetics were investigated at 40–60 °C and pH 10.0–12.0, and the plugging experiment was performed in low-permeability flooding CO_2_ cores at 50 °C.

### 2.1. Thermo-/pH-Dual-Sensitive Property

**Figure 1:**
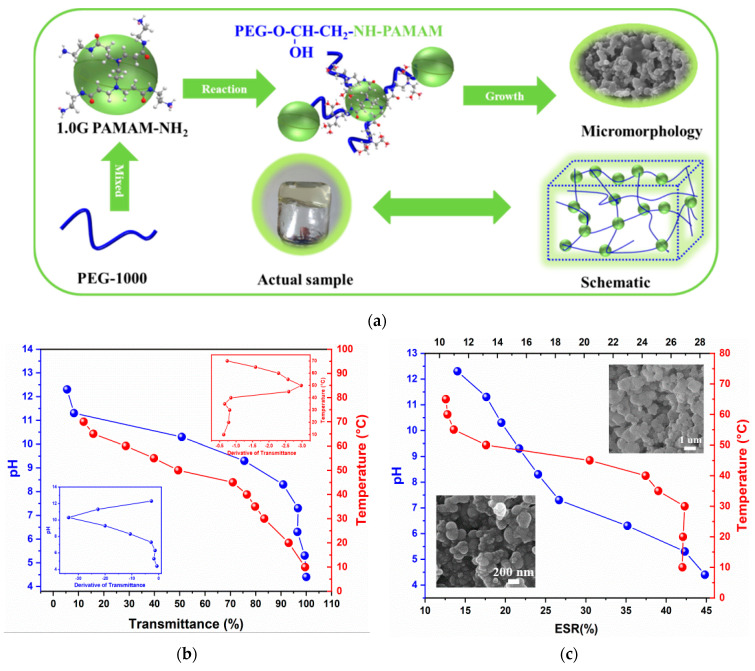
(**a**) Schematic diagram of PEG/PAMAM hydrogel gelation. (**b**) Transmittance and phase transition temperature and pH curves of PEG/PAMAM hydrogels. (**c**) Equilibrium swelling ratio curves of PEG/PAMAM hydrogels under different temperatures and pHs.

To tune the dual-sensitivity of the PEG/PAMAM hydrogels in the simulated Aonan block of Daqing oilfield, epichlorohydrin was employed as a linker between the PEG arms and the terminal -NH_2_ group in PAMAM with a salinity of 5103.2 mg/L. Additionally, PEG/PAMAM hydrogels were synthesized via ring-opening polymerization and crosslinking reactions among the -OH in PEG and the -NH_2_ in PAMAM and epichlorohydrin (Figure 1a). As is shown in Figure 1b,c, the transmittance and equilibrium swelling ratio present a similar variation tendency at different temperatures and pHs, respectively. In the pH range of 4.4–12.3, the transmittance decreases with an increase in the pH value; the derivative of transmittance was about pH 10.3 for the phase transition. Additionally, the equilibrium swelling ratio was found to decrease with an increase in pH, along with a decrease in the size of the gel from the micron (~1 um) to the nanometer scale (~200 nm). Generally, under acidic conditions, the nucleophile (-OH, -NH) attacks the side with more substituents on the epoxy group, and the carbon atom on the other side of the epoxy becomes a hydroxyl group. However, the nucleophile (-NH_2_) attacks the side of epoxy groups with few substituents under alkaline conditions, along with the regeneration of epoxy groups, leading to a lower gelation efficiency [30]. The transmittance and swelling properties suggest that the increase in temperature causes the volume of the hydrogels to shrink in the range of 10–70 °C and that the degree of change depends on the phase transition temperature, which was observed at 50 °C [31]. When the temperature is higher than 50 °C, hydrogel shrinks and loses water owing to the destruction of the physical crosslinking such as H-bonds, otherwise swelling and water absorption will occur. These observations indicate that PEG/PAMAM hydrogels exhibit thermo-/pH-dual-sensitive properties. Additionally, further characterizations should be performed to investigate the reaction kinetics and how to control the micro-size, morphology, and crosslinking density.

### 2.2. Reaction Dynamics

The gelation kinetics of the PEG/PAMAM hydrogels were investigated by the rheological method combined with the Avrami model and Dickinson model. Lam and Rogers concluded that, when the Avrami dynamics factor (*n*) is in the range of 1–1.7, the gel nucleates transiently and grows with a one-dimensional (1D) growth mode. Additionally, the gel exhibits a two-dimensional (2D) growth mechanism such as disk or plate when *n* is ~2 and a three-dimensional (3D) growth mode when *n* is ~3 or >3 [32]. In addition, the Dickinson dynamics density parameter of the dimension (D_d_) represents the gel aggregation state. If 1 < D_d_ < 1.5, the structural density of the gel is relatively loose. If 1.5 < D_d_ < 2, the gel has a medium aggregation state and density. If 2 < D_d_ < 3, the gel has a high aggregation state and density [33].

#### 2.2.1. Reaction Dynamics Studies on the Temperature Sensitivity of PEG/PAMAM Hydrogels

**Figure 2:**
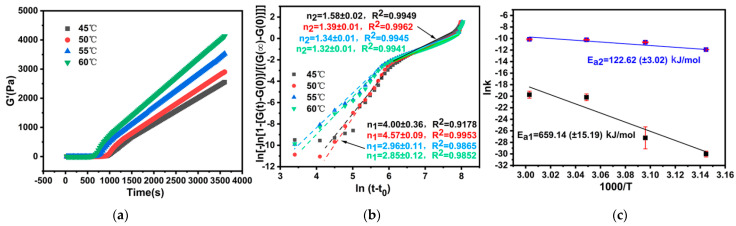
(**a**) Composite viscoelastic modulus. (**b**) Avrami dynamics model. (**c**) Activation energy during gelation at 4560 °C.

The variation tendency of the storage modulus was investigated during the gelation under the condition of 45–60 °C and pH 10.0. Figure 2 documents a secondary growth pattern from three to one dimension. Avrami dynamics factors of the secondary growth pattern were obtained. They are *n*_1_ = 4.00 and *n*_2_ = 1.58 at 45 °C, *n*_1_ = 4.57 and *n*_2_ = 1.39 at 50 °C, *n*_1_ = 2.96 and *n*_2_ = 1.34 at 55 °C, and *n*_1_ = 2.85 and *n*_2_ = 1.32 at 60 °C. The Avrami factor *n*_1_ was kept in the range of 2.85–4.57, and *n*_2_ was in the range of 1.32–1.58, indicating that the gel belonged to the 3D spherical nucleation mode, and then grew via a 1D growth mode at the temperature of 45–60 °C. The primary growth activation energy of the gel system is 659.14 kJ/mol, and the secondary growth activation energy is 122.62 kJ/mol. This indicates that the 3D spherical growth mode of the temperature-controlled gel requires more energy and the energy barrier of the gel reaction is higher, while the 1D mode of the secondary growth mode can gelatinize at a lower energy.

As is shown in Table 1, the gelation rate increased with an increase in temperature, but the primary 3D spherical growth rate of the system was slow (1 × 10^−11^–2.71 × 10^−8^), and the secondary 1D growth rate was accelerated (2.44 × 10^−5^–4.54 × 10^−4^), which made the spherical gel grow into a flake or ellipsoid shape. When the temperature reaches 60 °C, the growth mode transitions to a 2D–3D mixed mode, and irregular spherical gel is generated, which indicates that high temperature is not conducive to the formation of the spherical structure of the gel. The energy storage modulus of the system increases rapidly with an increase in temperature, indicating that the thermal motion of molecules accelerated the nucleation rate of the 3D gel structure. The growth mode changes after the gel reaches the saturation state, presenting a 1D mode of growth along the diameter, which stabilizes the sphere-like structure.

#### 2.2.2. Reaction Dynamics Studies on pH Sensitivity of PEG/PAMAM Hydrogels

**Figure 3:**
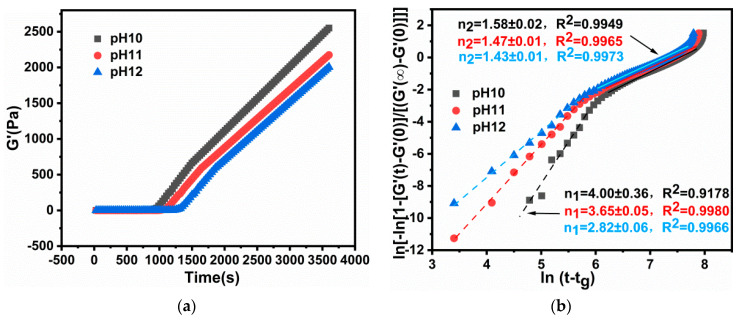
(**a**) Storage modulus. (**b**) Avrami dynamics model during gelation at pH 10.0 to 12.0.

The variation tendency of the storage modulus during gelation was observed at a pH range of 10.0–12.0 at 45 °C (Figure 3a). The secondary growth pattern exhibits a similar tendency from 3D to 1D. Owing to the phase transition being at pH 10.3, the Avrami dynamics factors decreased with an increase in pH; being about *n*_1_ = 4.00 and *n*_2_ = 1.58 at pH 10.0, *n*_1_ = 3.65 and *n*_2_ = 1.47 at pH 11.0, and *n*_1_ = 2.82 and *n*_2_ = 1.43 at pH 12.0. The Avrami factor *n*_1_ was in the range of 2.82–4.00, and 1.43–1.58 of *n*_2_, indicating that the gelation was still carried out with the spherical nucleation behavior and the secondary growth pattern at pH 10.0 to 12.0 (Figure 3b).

Moreover, the gelation rate also increased with an increase in pH, exhibiting a slow rate of 1 × 10^−11^–7.58 × 10^−8^ during the process of the primary 3D spherical growth and 2.44 × 10^−5^–2.76 × 10^−4^ at the secondary 1D growth stage, which made the spherical gel grow in a direction of a diametral plane (Table 2). However, the *n* value gradually decreased with an increase in pH, along with an accelerated speed of gelation. This suggests that higher pH can affect the growth of the 3D structure of the gel, but not the circular structure. When the pH was 12, the growth mode transformed into a 2D–3D mixed mode, generating an irregular spherical gel, which indicates that higher pH is not conducive to the formation of a spherical morphology but an ellipsoid or disk shape.

The results of thermo-/pH-dual-sensitivity illustrate that Avrami kinetic factor, gel reaction rate, and activation energy are all changed in the two growth stages, which are approximately consistent with the secondary nucleation patterns in 3D gel fiber networks [34]. Thus, the microstructure is related to the dense dimension of secondary growth in the process of dynamic gel reaction.

#### 2.2.3. Dickson’s Dynamics Density Dimension and Microstructure of PEG/PAMAM Hydrogels

Due to the special secondary growth mode of PEG/ PAMAM hydrogels, Dickinson’s quantitative methods should be employed to investigate the differences in gel aggregation state (D_d_) in the two growth stages under different temperatures and pHs and to obtain the microstructure of hydrogel during the reaction process.

**Figure 4:**
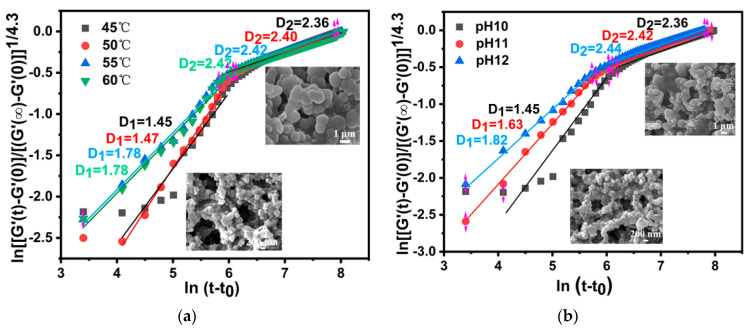
Relationship curves of dense dimension and microstructure of PEG/PAMAM hydrogels under different conditions. (**a**) Temperatures from 45 to 60 °C; (**b**) pH 10.0 to 12.0.

As is shown in Figure 4, in the process of the primary growth of gels, the density dimension of gels increases with an increase in temperature, and the D_d__1_ value is 1.45–1.78, indicating that the density of spherical gels is medium, and the spherical gel is relatively loose with a nanometer scale (~200 nm). Additionally, the D_d__2_ value then rises to 2.36–2.42, indicating that the compact dimension of the gel sphere is high, and the gel grows densely into an ellipsoid or disk shape with a micrometer scale (~1 μm). This illustrates that the kinetic density dimension of the PEG/PAMAM hydrogels increases with temperature. Furthermore, the D_d_ value exhibits a similar increased density dimension trend with an increase in pH, together with an irregular ellipsoid or disk shape. D_d_ is 1.45–1.82 in the process of primary growth and 2.36–2.44 in the secondary growth stage.

**Figure 5:**
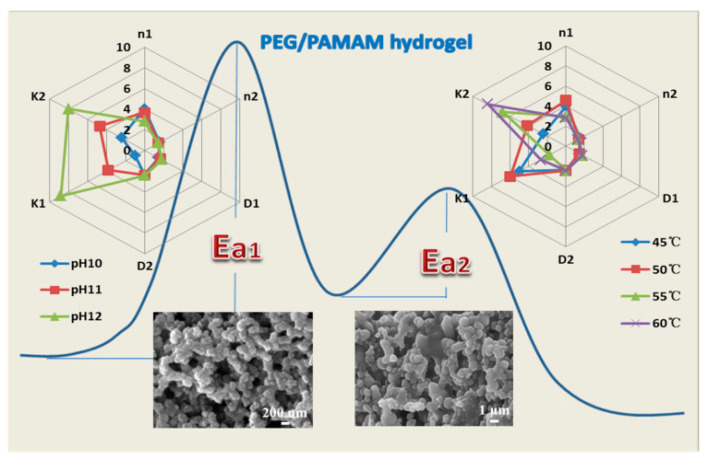
Schematic diagram of the two dynamic growth modes of PEG/PAMAM hydrogels.

As a result, PEG/PAMAM hydrogels present a secondary growth pattern from three dimensions to one dimension. High temperature and pH lead to an increase in the spherical aggregation of the hydrogel, along with an accelerated rate of gel reaction, leading to a scale transformation from micrometer to nanometer size, which is in agreement with the behavior of hydrogels whereby they shrink and lose water at temperatures higher than 50 °C and pHs higher than 10.0 (Figure 5).

### 2.3. Plugging Application of CO_2_ Channeling

In the Aonan block of Daqing oilfield, the reservoir depth is 1000–1400 m, the reservoir temperature is 45 to 60 °C, and the reservoir permeability is less than 50 × 10^−3^ μm^2^ [35]. In the process of CO_2_ flooding, the reservoir fracture is small (0.05–0.1 mm), and the micro pore throat radius is nano–micro size, which leads to a serious CO_2_ breakthrough problem. Moreover, pH decreases in the reservoir fluid during either CO_2_ flooding or CO_2_ storage. Thus, it is still a great challenge to develop a high-performance plugging agent and high-efficiency technology for CO_2_ channeling plugging in a complex environment. In this work, a thermo-/pH-dual-sensitive nanostructured PEG/PAMAM hydrogel was designed with the phase transition properties at 50 °C and pH 10.0 to overcome the size-matching difficulty, control the environmental sensitivity, and improve the plugging efficiency in low permeability reservoirs of CO_2_ flooding.

**Figure 6:**
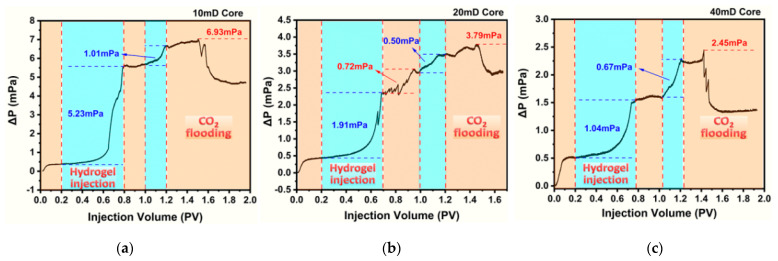
Plugging experiment with different matrix permeability cores under the environmental condition of 50 °C and pH 10.0. (**a**) 10 mD core, (**b**) 20 mD core, and (**c**) 40 mD core.

Three fractured cores (fracture width, 0.10 mm) with different matrix permeabilities were used to study the CO_2_ channel plugging. Plugging experiments were performed by alternating injections of CO_2_ and hydrogel solution in a slug type with a backpressure of 13 MPa under environmental conditions of 50 °C and pH 10.0, which is the phase transition point of the PEG/PAMAM hydrogel. Figure 6 documents a progressive upward tendency of the ΔP with the injection of CO_2_ and hydrogel until it reaches the breakout pressure. (1) In the process of the first CO_2_ flooding, the ΔP for the tree cores rises and then stabilizes at ~0.5 MPa. (2) In the process of the first hydrogel solution injection, ΔP increases greatly: 5.23 MPa for the 10 mD core, 1.91 MPa for the 20 mD core, and 1.04 MPa for the 40 mD core, indicating that it is more difficult to inject hydrogel solution into lower permeability cores. After gelation for 20 h, further CO_2_ flooding was implemented to investigate the environmental sensitivity and plugging of the PEG/PAMAM hydrogel. (3) In the process of the second CO_2_ flooding, the ΔP increases slightly in the 10 mD core and the 40 mD core, while the ΔP increases 0.72 MPa in the 20 mD core. These observations suggest that the PEG/PAMAM hydrogel shows environmental and CO_2_-responsive properties of swelling owing to the decreases in pH and temperature caused by CO_2_ flooding. Because the environmental sensitivity of the PEG/PAMAM hydrogel was inconspicuous in the 10 mD core and the 40 mD core, further hydrogel solution injection was implemented. (4) In the process of the second hydrogel solution injection, the ΔP still increase by 1.01 MPa for the 10 mD core, 0.50 MPa for the 20 mD core, and 0.76 MPa for 40 mD core, illustrating that the injected volume of the hydrogel solution does not reach saturation. (5) In the process of the third CO_2_ flooding, the ΔP exhibited further increases. The breakthrough pressure reaches 19.93 MPa for the 10 mD core, 16.76 MPa for the 20 mD core, and 15.45 MPa for the 40 mD core, respectively (Table 3). The pressure discrepancy may result from the difference in the microcosmic pore radius and distribution in the core. In addition, our previous work concluded that the size of gel particles is the decisive factor in plugging. Relatively large nano-sized gels have a strong blocking channel ability to balance the pressure and coordinate the effective fluid flow path [36]. Thus, in the 20 mD core, the PEG/PAMAM hydrogel can exert its best environmental response, CO_2_ response, and plugging ability.

Moreover, the corresponding residual pressures remain at 17.64 MPa, 15.91 MPa, and 14.34 MPa, along with plugging efficiencies of 99.386%, 99.425%, and 99.258%, respectively, showing great plugging performance and having high residual resistance after being broken through by CO_2_ (Table 3). These results demonstrate that the PEG/PAMAM nano hydrogel can increase the seepage resistance in fractures. It is a good environmental response candidate for plugging applications of CO_2_ channeling in low-permeability cores.

## 3. Conclusions

A thermo-/pH-dual-sensitive nanostructured PEG/PAMAM hydrogel was employed to research the reaction dynamics via the rheological method combined with the Avrami and Dickinson dynamics models for application in plugging CO_2_ channeling in low-permeability cores.

(1) PEG/PAMAM hydrogels present a secondary growth pattern from three to one dimension. The phase transition temperature is 50 °C, and the phase transition pH is 10.0.
(2) PEG/PAMAM hydrogels exhibit a secondary growth pattern from three to one dimension. The primary growth activation energy of the gel system is 659.14 kJ/mol, and the secondary growth activation energy is 122.62 kJ/mol under the condition of 45–60 °C and pH 10.0.
(3) High temperature and pH lead to an increase in the spherical aggregation of the hydrogel, along with an accelerated rate of gel reaction, leading to a scale transformation from micrometer to nanometer size and a morphology transformation from a sphere to an irregular ellipsoid or disk shape.
(4) PEG/PAMAM hydrogels present great plugging performance (plugging efficiency > 99%) and have high residual resistance after being broken through by CO_2_ in low-permeability cores.

It is a good environmental response and CO_2_-responsive candidate for plugging applications. However, the in situ controlling of the gelation time and strength, the controllable environmental responsive plugging and unplugging, and the optimized injection technology remain to be studied. Thus, it can not only increase the CO_2_ sweep efficiency and enhance oil recovery but can also capture and bury CO_2_ to achieve the goal of carbon neutrality in oil fields.

## 4. Materials and Methods

### 4.1. Materials

Thermo-/pH-dual-sensitive nanostructured PEG/PAMAM hydrogels were synthesized by the same method reported in our previous work [29]. All the chemicals (1.0G PAMAM (>99.8%), PEG (Mn = 1000, >99.5%), epichlorohydrin (>99.5%), tetrabutylammonium bromide (>99.8%), sodium hydroxide (AR), and anhydrous ethanol) were purchased from Aladdin Reagent Co., Ltd. (Shanghai, China). Deionized water was made in our laboratory.

Artificial cores synthesized with quartz sand and epoxy resin were employed to perform the plugging experiments. The physical properties of the artificial cores are listed in Table 4. CO_2_ with a purity of 99.99% was supplied by Daqing Special Gas Co., Ltd. Additionally, the injection water was simulated formation water with a pH of 8.1. The composition of the simulated formation water is shown in Table 5.

### 4.2. Methods

#### 4.2.1. Avrami Reaction Dynamics Model Based on Rheology

A combination method of rheology and the Avrami dynamics model was established to investigate the dynamic gelation process of thermo-/pH-dual-sensitive PEG/PAMAM nanogels and define the microstructure of gelation.

Viscosity is an important parameter to judge whether a gel system is formed. Therefore, the equilibrium state of gelation was qualitatively determined by the change of viscosity over time.

P(t) = (η_t_ − η_0_)/ (η_b_ − η_0_) (1)

where, P(t) is the gel volume fraction, η_0_ is the initial viscosity, η_b_ is the equilibrium viscosity of the gel, and η_t_ is the viscosity at any given time. The viscosity unit is mPa·s.

The viscosity and rheology of the system are both functions of gelation over time.

η_t_ = G’/ω (2)

where, G’ is the storage modulus and ω is the shear rate.

During the gelation process, the gel volume fraction P(t) is the gelation ratio in the Avrami dynamics model. Thus, the Avrami dynamics model was updated.

P(t) = 1 − exp(−*kt*^n^) (3)

where, *k* is the kinetic constant, n is the Avrami factor, and *t* is the reaction time.

By combining Equations (1)–(3) and taking the natural logarithm of both ends of the formula, we obtained the relationship between the Avrami dynamics model and rheology [37]:

ln {−ln[1 − (G’(*t*) − G’(*0*))/(G’(*b*) − G’(*0*))]}  = *n*ln(*t* − *t*_0_) + ln*k* (4)

where, *t*_0_ is the initial time of gel nucleation. In terms of the nucleation rate and growth rate, *k* is the gelation rate constant. *n* is the Avrami factor, which represents the nature of gel nucleation and the growth mode of the gel structure. The Avrami parameters *k* and *n* can be obtained from the linear intercept and slope, respectively.

The effect of temperature on the reaction kinetics can be calculated by the combination of the Arrhenius equation and the updated Avrami kinetic model, which allows us to obtain the relationship between the nucleation rates, growth mode, and activation energy of the gel.

ln {−ln[1 − (G’(*t*) − G’(*0*))/(G’(*b*) − G’(*0*))]}  = *n*ln(*t* − *t*_0_) + ln*k* − Ea/RT (5)

Mathematically, the intercept of Equation (5) is equal to ln*k* − Ea/RT. The reaction rate of the gel is linearly related to the reaction temperature, so the activation energy of the gel can be calculated in a certain temperature range.

#### 4.2.2. Dickinson Reaction Dynamics Model Based on Rheology

The Dickinson quantitative method was used to study the change of the storage modulus during the dynamic reaction of the gel, giving us the difference in the growth mode and the microstructure of the hydrogel.

The Dickinson formula:

P(t)∝*k*·*t* ^(3−D^_d_^)/D^_d_ (6)

where, D_d_ is the dynamics density parameter of dimension. The proportionality factor is set as θ and named the scale factor. Equations (1) and (2) combined with Equation (1) give us Equation (7):

ln {(G’(*t*) − G’(*0*))/(G’(*b*) − G’(*0*))} ^1/^^θ^ = ((3 − D_d_)/D_d_) ln(*t* − *t*_0_) + ln*k* (7)

In the process of gel generation, if there are different growth modes with an inflection point, different growth modes can be divided according to the inflection point. Additionally, the scaling factor can be calculated. Generally, θ is 4.2 for the spherical gel [38]. The density dimension parameters of gel D_d_ in the different growth processes represent the gel microstructure during the dynamic gelation process.

#### 4.2.3. Plugging Experiment

**Figure 7:**
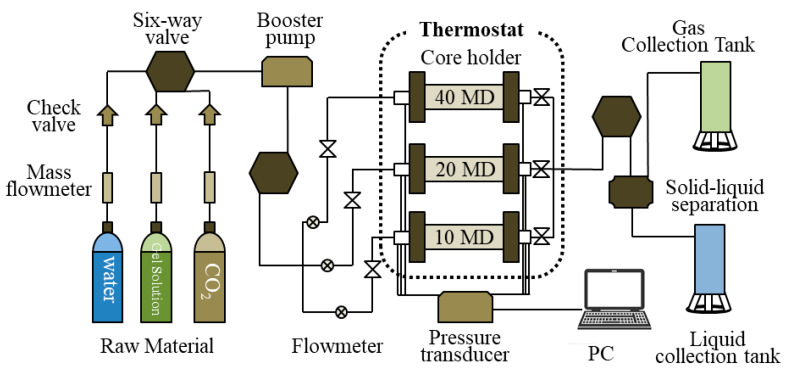
Schematic diagram of the plugging device.

Plugging experiments were performed by alternating injections of CO_2_ and hydrogel solution in a slug type on three fractured low permeability cores in a parallel manner at 50 °C (Figure 7). A schematic of the plugging experiment is shown in Figure 7. The detailed experimental procedures were as follows: (1) Simulated formation water was injected at 0.5 mL/min until the pressure difference across the core holder was stable. (2) The first CO_2_ flooding was carried out at 0.5 mL/min for 0.2 pore volume (PV). Additionally, the back pressure was set to 13.0 MPa. (3) The first hydrogel solution (8 wt%) was injected into the cores to form plugs at a speed of 0.05 mL/min. The injection was stopped when the pressure difference was not changed. (4) After 20 h, the second CO_2_ flooding was implemented at 0.5 mL/min. (5) The second hydrogel solution was injected with the same at 0.05 mL/min for about 0.2 PV. (6) After 20 h, further CO_2_ flooding was injected at 0.5 mL/min until the residual pressure difference was not changed. During the whole process, the confining pressure was maintained at 2 MPa higher than the injection pressure, and the core was closed at the end of each step.

### 4.3. Characterizations

The phase transition temperature and pH were observed using a TU-1901 ultraviolet-visible spectrophotometer (UV-Vis, Yixin Instrument Equipment Co. Ltd., China). The determination wavelength was fixed at 210 nm. The hydrogel morphology was observed using an S-4700 cold-field scanning electron microscope (SEM, Hitachi, Tokyo, Japan) with a 20 kV accelerating voltage. The rheological properties of the PEG/PAMAM nanogels were measured on a controlled-stress rheometer (AR2000, TA Instruments, New Castle, DE, USA), equipped with a closed container to minimize evaporation. Dynamic oscillatory tests at a frequency of 1 Hz were performed to measure the storage and loss moduli with a shearing rate of 6.28 s^−1^ at 50 °C. The equilibrium swelling of the hydrogel was calculated according to Buwalda’s report [12]. The injections of CO_2_ and hydrogel solution experiments were performed on a YXQT-1 type core displacement device (Hua ‘an Scientific Research Instrument Co. Ltd., Jiangsu, China). Core permeability was calculated using Darcy’s law:

Q/S = −10K·ΔP/ηL(8)

where, Q is the flow of carbon dioxide (cm^3^/s), S is the cross-sectional area of the core (cm^2^), K is permeability (μm^2^), ΔP is the pressure difference between both ends of the core (MPa), η is CO_2_ fluid viscosity (mPa·s), and L is the core length (cm).

## Figures and Tables

**Table 1:** Gelation kinetics parameters of PEG/PAMAM hydrogels (45–60 °C).

Temperature (°C)	*n*_1_	Growth Pattern	*n*_2_	Growth Pattern	*k*_1_	*k*_2_	Ea_1_ (kJ/mol)	Ea_2_ (kJ/mol)
45	4.00	3D	1.58	1D	1.00 × 10^−11^	2.44 × 10^−5^	659.14	122.62
50	4.57	3D	1.39	1D	2.00 × 10^−11^	6.15 × 10^−5^		
55	2.96	3D	1.34	1D	1.74 × 10^−8^	3.68 × 10^−4^		
60	2.85	2D–3D	1.32	1D	2.71 × 10^−8^	4.54 × 10^−4^		

**Table 2:** Gelation kinetics parameters of PEG/PAMAM hydrogels (pH 10.0–12.0).

pH	*n*_1_	Growth Pattern	*n*_2_	Growth Pattern	*k*_1_	*k*_2_
10.0	4.00	3D	1.58	1D	1 × 10^−11^	2.44 × 10^−5^
11.0	3.65	3D	1.47	1D	3.8 × 10^−10^	1.67 × 10^−4^
12.0	2.82	2D–3D	1.43	1D	7.58 × 10^−8^	2.76 × 10^−4^

**Table 3:** Results of the plugging experiments.

Core	Matrix Permeability (mD)	Fracture Permeability (mD)	Plugging Efficiency (%)	Breakthrough Pressure (MPa)	Residual Pressure (MPa)
1	10.378 mD	0.0637	99.386	19.93	17.64
2	20.247 mD	0.1164	99.425	16.79	15.91
3	40.183 mD	0.2981	99.258	15.45	14.34

**Table 4:** Physical parameters of the artificial cores.

Core	Permeability (mD)	Porosity (%)	Length (cm)	Diameter (cm)	Fracture Width (mm)
1	10.378 mD	7.93	10.0	2.5	0.1
2	20.247 mD	9.79	10.0	2.5	0.1
3	40.183 mD	12.45	10.0	2.5	0.1

**Table 5:** Composition of simulated formation water (mg/L).

Ion Type	K^+^ + Na^+^	Ca^2+^	Mg^2+^	SO_4_^2−^	Cl^−^	HCO_3_^−^	Salinity
	2036.5	316.2	119.5	159.8	809.2	1641.4	5103.2

## Data Availability

All the persons included have agreed to confirm.
